# The genetic effect of the *ICAM1* (intercellular adhesion molecule 1) rs5498 polymorphism on the susceptibility towards multiple sclerosis

**DOI:** 10.1042/BSR20181642

**Published:** 2018-12-11

**Authors:** Chuan Jiang, Chunli Xie, Jianli Feng, Maolin Hao

**Affiliations:** 1 Department of Neurology, Shandong Provincial Hospital Affiliated to Shandong University, Jinan, Shandong 250022, P.R. China; 2 Department of Neurology, Fourth People’s Hospital of Jinan, Jinan, Shandong 250031, P.R. China

**Keywords:** ICAM1, multiple sclerosis, rs5498, risk

## Abstract

In the present study, we included currently published evidence to comprehensively evaluate the influence of the rs5498 polymorphism within the *ICAM1* (intercellular adhesion molecule 1) gene on the genetic risk of multiple sclerosis. STATA 12.0 software was utilized to carry out the heterogeneity assessment, association test, and Begg’s test as well as the Egger’s tests and sensitivity analyses. A total of 11 high-quality case–control studies were selected from the initially retrieved 2209 articles. The lack of high heterogeneity led to the use of a fixed-effect model in all genetic models. The results of the association test showed a reduced risk of multiple sclerosis in the allelic G vs A (*P*_association_ = 0.036, OR = 0.91) and dominant AG+GG vs AA (*P*_association_ = 0.042, OR = 0.85) but not in other genetic models (all *P*_association_ > 0.05). In addition, the negative results were observed in further subgroup analyses based on ethnicity or Hardy-Weinberg equilibrium in all genetic models. Data from Begg’s and Egger’s tests further excluded the presence of remarkable publication bias, while sensitivity analysis data supported stable outcomes. Thus, we conclude that *ICAM1* rs5498 may not be related to the risk of multiple sclerosis in Caucasian or Asian populations, which still merits further research.

## Background

Multiple sclerosis is a type of chronic degenerative disease in the central nervous system (CNS) with the features of inflammatory demyelination-induced relapses and progressive neurological disability [1,2]. Multiple sclerosis (MS) mainly contains four phenotypic classifications, namely, relapsing-remitting MS (RRMS), primary progressive MS (PPMS), secondary progressive MS (SPMS), and clinically isolated syndrome (CIS) [3]. As a common autoimmune disease with neurodegeneration, immunological and genetic factors were involved in the initiation and development of multiple sclerosis [4–9]. For instance, as a primary risk allele, HLA DRB1*15 (HLA class II histocompatibility antigen, DRB1 beta chain *15) within the major histocompatibility complex (MHC) was associated with the susceptibility to multiple sclerosis [4]. A number of genetic variants, such as *IL7R* (interleukin 7 receptor) rs6897932, *IL2RA* (interleukin 2 receptor subunit alpha) rs2104286 and *CD58* (cluster of differentiation 58) rs2300747 polymorphism, may be linked to the risk and progression of multiple sclerosis based on the data of large genome-wide association studies [5]. However, no specific molecular mechanism underlying the clinical course of multiple sclerosis has been uncovered. Herein, we aimed to comprehensively estimate the possible genetic impacts of the rs5498 polymorphism of the *ICAM1* (intercellular adhesion molecule 1) gene on the susceptibility to multiple sclerosis.

The ICAM-1 protein, which is also referred to as CD54 (cluster of differentiation 54), participates in a group of biological processes regarding the immune responses [10]. As a member of the immunoglobulin superfamily, some membrane-bound and soluble ICAM-1 isoforms exist [10]. Abnormal cell surface ICAM-1 expression status and soluble ICAM-1 level are related to the pathogenesis of some clinical diseases (e.g. multiple sclerosis, asthma, rhinitis [11–13] etc).

Several polymorphic variants, such as rs5498 A/G (Lys469Glu), rs1799969 G/A (Gly241Arg) and rs5491 A/T (Lys56Met), were reported within the *ICAM1* gene on chromosome 19 [14]. Several meta-analyses showed the different role of *ICAM1* polymorphisms in some clinical diseases. For example, the *ICAM1* rs5498 polymorphism was reported to be linked to a decreased risk of myocardial infarction (MI) [15]. The *ICAM1* rs1799969 polymorphism may be associated with the occurrence of Bechet’s disease (BD), rheumatoid arthritis (RA) [16], and cancer [17]. As far as we know, two prior meta-analyses from 2000 [18] and 2003 [19] evaluated the association of *ICAM1* rs5498 with the risk of multiple sclerosis; however, different conclusions were obtained. We therefore performed an updated meta-analysis for a comprehensive reassessment based on the available data.

## Materials and methods

### Database search

**Figure 1:**
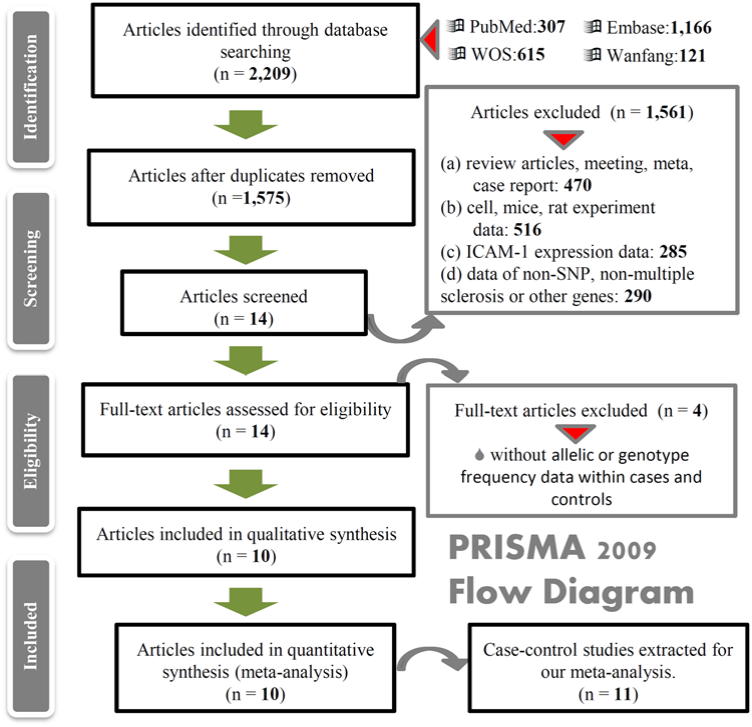
The selection process of eligible case–control studies

We designed and performed our meta-analysis prior to September 2018, following the preferred reporting items for systematic reviews and meta-analyses (PRISMA). The PRISMA-based analysis process was depicted in Figure 1. Four online databases, namely, PubMed, Embase, Web of Science (WOS) and Wanfang, were employed. The different terms ‘ICAM1’ and ‘multiple sclerosis’ were combined. The search terms in PubMed were as follows: (((((((Multiple Sclerosis) or Sclerosis, Multiple) or Sclerosis, Disseminated) or Disseminated Sclerosis) or MS (Multiple Sclerosis)) or Multiple Sclerosis, Acute Fulminating)) and (((((((Intercellular Adhesion Molecule 1) or Intercellular Adhesion Molecule 1) or ICAM1) or CD54 Antigens) or CD54 Antigen) or Antigen, CD54) or Antigens, CD54).

### Article screening

Adhering to the inclusion and exclusion criteria, we carefully screened the articles and finally evaluated the eligible case–control studies. Inclusion criteria: (a) case and control studies; (b) multiple sclerosis; (c) rs5498 polymorphism within the *ICAM1* gene; and (d) allelic or genotype frequency data within cases and controls. Exclusion criteria: (a) review articles, meeting, meta, case report; (b) cell, mice, rat experiment data; (c) ICAM-1 expression data; and (d) data of non-SNP, non-multiple sclerosis or other genes.

### Data collection

Of the eligible case–control studies, the first author, publication time, ethnicity, polymorphism, genotype frequency of control and case, control source, genotyping method, sample size, and *P* value of Hardy–Weinberg equilibrium were collected and summarized in the tables. The study quality was appraised using the system of NOS (Newcastle–Ottawa quality assessment Scale).

### Statistical methods

Statistical analysis was conducted with STATA 12.0 (Stata Corporation, College Station, TX, U.S.A.). Briefly, we performed the *I*^2^-test and *Q*-statistical test to check the statistical heterogeneity of the studies. High heterogeneity (*I*^2^ > 50% or *P* < 0.05) led to the utilization of the DerSimonian and Laird method under the random-effect model, whereas the absence of remarkable heterogeneity (*I*^2^ < 50% or *P* > 0.05) led to the use of the Mantel–Haenszel method under a fixed-effect model.

Additionally, we computed the OR (odds ratio), 95% CI (95% confidence interval), and *P* value from the association tests under the allelic, homozygote, heterozygote, dominant, recessive, and carrier models, respectively. We performed the subgroup analyses based on the factors of ethnicity or Hardy–Weinberg equilibrium. We also used Begg’s test and Egger’s test to evaluate the potential publication bias. Sensitivity analysis with the sequential omission of each study was adopted as well to test the data stability.

## Results

### Eligible case–control studies

After the computerized retrieval of databases, a total of 2209 articles (PubMed with 307 articles, Embase with 1166 articles, WOS with 615 articles and Wanfang with 121 articles) were obtained. We then discarded 634 duplicates and 1561 articles depending on the exclusion criteria (shown in Figure 1). Another four articles without the allelic or genotype frequency data in case or control groups were ruled out. Consequently, we obtained a total of 11 case–control studies from ten articles [18–27] for the summarization of basic information. As listed in Table 1, all studies utilized population-based controls and showed high quality for analysis, in that the score of the NOS system in each study was greater than five.

**Table 1:** Basic information of the studies included in the meta-analysis

First author [reference]	Year	Region	Ethnicity	NOS	AA/AG/GG (control)	χ^2^	*P*_HWE_	AA/AG/GG (case)	Methods
**Killestein** [18]	**2000**	Netherlands	Caucasian	5	40/43/23	2.97	0.09	42/68/35	NR
**Li** [20]	**2008**	China	Asian	8	35/17/3	0.24	0.63	29/16/6	PCR-LDR
**Luomala** [21]	**1999**	Finland	Caucasian	7	27/59/25	0.45	0.50	34/45/25	PCR-RFLP
**Marrosu** [22]	**2000**	Sardinia	Caucasian	8	44/59/23	0.17	0.68	49/75/33	PCR
**Mousavi** [23]	**2007**	Iran	Asian	7	34/80/42	0.13	0.72	45/75/37	PCR
**Mycko** [24]	**1998**	Poland	Caucasian	6	23/20/25	11.50	<0.05	42/23/14	PCR
**Nejentsev** [19]	**2003**	Finland	Caucasian	6	146/302/125	1.77	0.18	92/119/66	PCR-RFLP
		Spain	Caucasian	6	33/47/33	3.19	0.07	49/55/36	PCR-RFLP
**Qiu** [25]	**2013**	Australia	Caucasian	7	318/678*	–	>0.05	240/460*	TaqMan
**Sanadgol** [26]	**2011**	Iran	Asian	7	69/36/18	10.57	<0.05	49/24/5	PCR-SSP
**Shawkatova** [27]	**2017**	Slovakia	Caucasian	8	68/101/39	0.02	0.89	75/133/40	PCR-RFLP

Abbreviations: HWE, Hardy–Weinberg equilibrium; LDR, ligase detection reaction; NOS, Newcastle–Ottawa Scale; NR, not reported; PCR, polymerase chain reaction; RFLP, restriction fragment length polymorphism; SSP, sequence-specific primers; *, the allelic frequency data of A/G; –, no data.

### Meta-analysis result

As shown in Table 2, 11 studies comprising 1786 cases and 2137 controls were included in the meta-analysis of allelic G vs A models, while ten case–control studies (1436/1639) were used for other genetic models. The lack of high inter-study heterogeneity (all *P*_heterogeneity_ > 0.05 and *I*^2^ < 50%) led us to use the fixed-effect model for all genetic models. After pooling the different studies together, we observed a reduced risk of multiple sclerosis in the allelic G vs A (Table 2, *P*_association_ = 0.036, OR = 0.91) and dominant AG+GG vs AA (*P*_association_ = 0.042, OR = 0.85) but not in other genetic models (all *P*_association_ > 0.05), thereby suggesting that *ICAM1* rs5498 is not a strong susceptibility locus for multiple sclerosis in the whole population.

**Table 2:** Meta-analysis of *ICAM1* rs5498 and multiple sclerosis risk

Genetic model	Sample size		Association analysis		Heterogeneity assessment	
	Study	Case/control	*P*_association_	OR (95% CI)	*P*_heterogeneity_	*I*^2^
**Allelic G vs A**	11	1786/2137	**0.036**	0.91 [0.83–0.99]	0.051	45.1%
**GG vs AA**	10	1436/1639	0.070	0.83 [0.68–1.02]	0.104	38.1%
**AG vs AA**	10	1436/1639	0.086	0.86 [0.73–1.02]	0.147	32.6%
**AG+GG vs AA**	10	1436/1639	**0.042**	0.85 [0.73–0.99]	0.070	43.2%
**GG vs AA+AG**	10	1436/1639	0.416	0.93 [0.78–1.11]	0.173	29.5%
**Carrier G vs A**	10	1436/1639	0.287	0.94 [0.83–1.06]	0.660	0.0%

Abbreviations: CI, 95% confidence interval; OR, odds ratio.

### Subgroup analysis results

**Figure 2:**
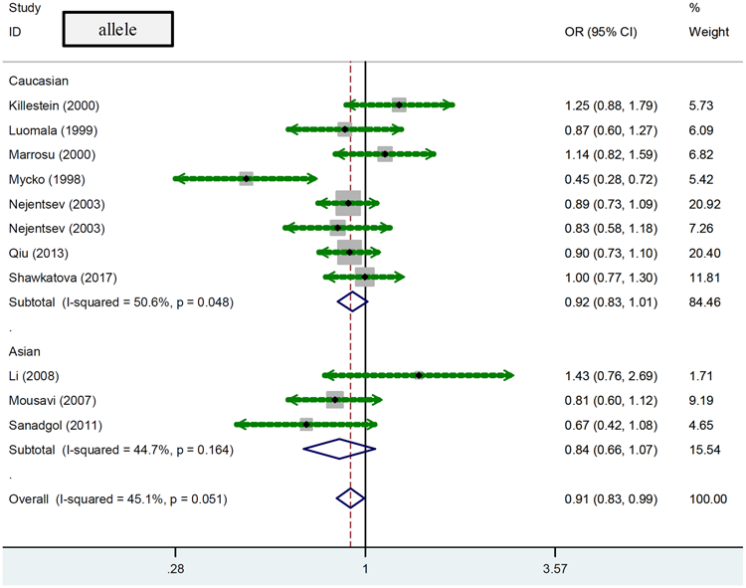
Forest plot of subgroup meta-analysis by ethnicity under the allelic model

We also conducted a series of subgroup analyses based on the factors of ethnicity or Hardy–Weinberg equilibrium. As shown in Table 3, eight case–control studies (1500/1803) were included for the subgroup analysis of ‘Caucasian’ under the allelic model, while seven case–control studies (1150/1305) were enrolled for other genetic models. There was no statistically significant difference between cases and controls in Caucasian populations (Table 3, *P*_association_ > 0.05). Similarly, no significant association between *ICAM1* rs5498 and multiple sclerosis was observed in subgroup analyses of studies with Asian populations or studies with *P*_HWE_ > 0.05 under any of the genetic models (Table 3, all *P*_association_ > 0.05). Forest plots of subgroup meta-analysis by ethnicity are shown in Figure 2 (allele), Supplementary Figure S1 (homozygote), Supplementary Figure S2 (heterozygote), Supplementary Figure S3 (dominant), Supplementary Figure S4 (recessive), and Supplementary Figure S5 (carrier). As a consequence, *ICAM1* rs5498 may not be associated with the risk of multiple sclerosis in Caucasian or Asian populations.

**Table 3:** Subgroup analysis of *ICAM1* rs5498 and multiple sclerosis risk

Genetic model	Subgroup	Sample size		Association analysis	
		*N*	Case/control	*P*_association_	OR [95% CI]
**Allelic G vs A**	Caucasian	8	1500/1803	0.094	0.92 [0.83–1.01]
	Asian	3	286/334	0.155	0.84 [0.66–0.99]
	*P*_HWE_ > 0.05	9	1629/1946	0.253	0.95 [0.86–1.04]
**GG vs AA**	Caucasian	7	1150/1305	0.194	0.86 [0.69–1.08]
	Asian	3	286/334	0.131	0.68 [0.42–1.12]
	*P*_HWE_ > 0.05	8	1279/1448	0.460	0.92 [0.74–1.14]
**AG vs AA**	Caucasian	7	1150/1305	0.132	0.87 [0.72–1.04]
	Asian	3	286/334	0.407	0.86 [0.59–1.24]
	*P*_HWE_ > 0.05	8	1279/1448	0.131	0.87 [0.73–1.04]
**AG+GG vs AA**	Caucasian	7	1150/1305	0.093	0.86 [0.72–1.03]
	Asian	3	286/334	0.236	0.81 [0.58–1.15]
	*P*_HWE_ > 0.05	8	1279/1448	0.190	0.89 [0.76–1.06]
**GG vs AA+AG**	Caucasian	7	1150/1305	0.682	0.96 [0.79–1.17]
	Asian	3	286/334	0.293	0.80 [0.52–1.22]
	*P*_HWE_ > 0.05	8	1279/1448	0.857	1.02 [0.84–1.23]
**Carrier G vs A**	Caucasian	7	1150/1305	0.427	0.95 [0.83–1.08]
	Asian	3	286/334	0.422	0.89 [0.68–1.18]
	*P*_HWE_ > 0.05	8	1279/1448	0.660	0.97 [0.86–1.10]

Abbreviations: CI, 95% confidence interval; HWE, Hardy–Weinberg equilibrium; *N*, number of studies; OR, odds ratio.

### Publication bias and sensitivity analysis

**Figure 3:**
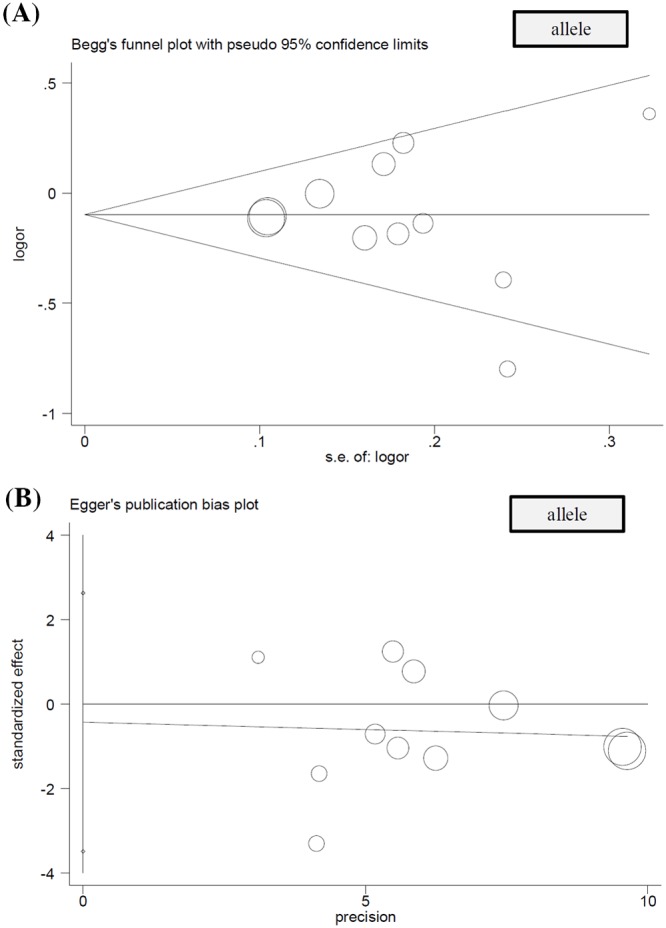
Publication bias analysis under the allelic model (**A**) Begg’s test and (**B**) Egger’s test.

**Figure 4:**
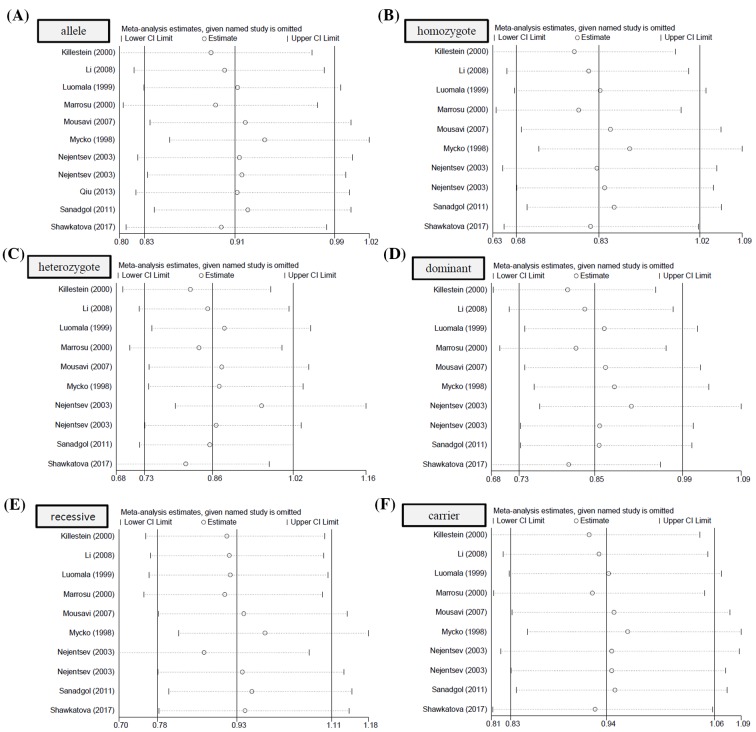
Sensitivity analysis data (**A**) allelic model, (**B**) homozygote model, (**C**) heterozygote model, (**D**) dominant model, (**E**) recessive model, and (**F**) carrier model.

As shown in Table 4, no large publication bias was detected in all the above comparisons (all *P*_Begg_ > 0.05, *P*_Egger_ > 0.05). Publication bias plots were presented in Figure 3 (allele), Supplementary Figure S6 (homozygote), Supplementary Figure S7 (heterozygote), Supplementary Figure S8 (dominant), Supplementary Figure S9 (recessive), and Figure S10 (carrier). The relatively stable or credible outcomes were also observed in our sensitivity analyses under all genetic models (Figure 4A–F).

**Table 4:** Assessment of publication bias

Genetic model	Begg’s test^*^		Egger’s test	
	*P*_Begg_	*z*	*P*_Egger_	*t*
**Allelic G vs A**	0.755	0.31	0.758	-0.32
**GG vs AA**	1.000	0.00	0.867	-0.17
**AG vs AA**	0.858	0.18	0.577	0.58
**AG+GG vs AA**	0.474	0.72	0.825	0.23
**GG vs AA+AG**	1.000	0.00	0.442	-−0.81
**Carrier G vs A**	0.858	0.18	0.661	-0.46

*, continuity corrected.

## Discussion

During the systematic database searches, we evaluated the inconclusive or controversial results on the genetic influence of the *ICAM1* rs5498 polymorphism in the occurrence of multiple sclerosis. For example, the ‘G/G’ genotype of the *ICAM1* rs5498 polymorphism was reportedly associated with a reduced risk of multiple sclerosis patients in Poland [24] rather than Finland [21]. Additionally, the *ICAM1* rs5498 polymorphism was not related to the multiple sclerosis risk in Dutch [18] or Iranian [23,26] populations or in the Han nationality of Henan, China [20]. There is also no statistically significant difference in *ICAM1* rs5498 frequencies between multiple sclerosis cases and negative control in the Slovak population [27]. However, the ‘G/G’ genotype may be associated with the development of multiple sclerosis at an earlier age [27]. These data deserve quantitative synthesis and a comprehensive assessment of the role of the *ICAM1* rs5498 polymorphism in the susceptibility to multiple sclerosis.

In 2000, Killestein, J. enrolled three case–control studies [18,21,24] to conduct the first meta-analysis by the Mantel–Haenszel method and did not detect the genetic role of *ICAM1* rs5498 polymorphism in the risk of multiple sclerosis [18]. In 2003, Nejentsev, S. et al. performed another meta-analysis, which involved five studies [18,19,21,22,24], and reported that the ‘A/A’ genotype of *ICAM1* rs5498 may be associated with an enhanced susceptibility to multiple sclerosis [19]. In the present study, we obtained a total of 11 case–control studies from ten eligible articles and performed another updated meta-analysis and the following stratification analysis by ethnicity and Hardy–Weinberg equilibrium, under the models of allelic G vs A, GG vs AA (homozygote), AG vs AA (heterozygote), AG+GG vs AA (dominant), GG vs AA+AG (recessive), and carrier G vs A. Even though a weakly significant difference between multiple sclerosis cases and controls was observed in the overall meta-analysis under the allele and dominant models, no significant association between *ICAM1* rs5498 and multiple sclerosis risk was detected in other inheritance models (homozygote, heterozygote, recessive, and carrier) of the overall meta-analysis and in neither of the inheritance models used in subgroup analyses. Hence, our data failed to support a strong connection between *ICAM1* rs5498 polymorphism and the susceptibility to multiple sclerosis, which is in line with the conclusion of prior pooling analysis [18].

Several highlights exist in our study. First, population-based negative controls were utilized in all the eligible studies. Second, the results of Begg’s and Egger’s tests excluded the presence of large publication bias. Third, there is no evidence of high heterogeneity between studies in all meta-analyses. Fourth, stable results were detected in our sensitivity analyses. In addition, it is worth mentioning that, during the extraction of the genotype frequency data in the report by Luomala et al. in 1999 [21], we utilized the updated data in 2000 [18].

Nonetheless, we still need to note some disadvantages, which may affect our statistical power. First, as in other meta-analyses, small sample size was a factor in some comparisons. For instance, from 2209 articles, only eleven case–control studies were selected for statistical analysis, and only three case–control studies [20,23,26] were enrolled in the subgroup analysis of ‘Asian’. Less than ten case–control studies were enrolled in the subgroup analysis of ‘Caucasian’. Second, the genotype distribution of control groups in two studies [24,26] deviated from Hardy–Weinberg equilibrium. As a result, no significant association between *ICAM1* rs5498 and multiple sclerosis risk was detected in the meta-analysis when only case–control studies with *P*_HWE_ > 0.05 were enrolled. Third, of the eligible case–control studies, only allelic frequency data were extracted in one study [25]. Fourth, we only investigated the impact of the *ICAM1* rs5498 polymorphism in the susceptibility to multiple sclerosis. Very limited data resulted in the failure of the relative meta-analysis concerning the combination of *ICAM1* rs5498 and other potential functional variants, such as rs1799969 G/A polymorphism. Fifth, we should consider more adjusted factors (e.g. age, sex, exposure, clinical characteristic, or pharmacotherapy et al.) in the future, when more usable evidence is available.

In conclusion, we incorporated the current data for an updated pooling analysis, which indicated that the *ICAM1* rs5498 polymorphism is not linked to the risk of multiple sclerosis in Caucasian and Asian populations. Given the limitations of our study, this negative conclusion still needs to be confirmed by more available evidence.

## Supporting information

**Figure S1:** 

**Figure S2:** 

**Figure S3:** 

**Figure S4:** 

**Figure S5:** 

**Figure S6:** 

**Figure S7:** 

**Figure S8:** 

**Figure S9:** 

**Figure S10:** 

## Abbreviations

95% CI: 95% confidence interval
BD: Bechet’s disease
CD54: cluster of differentiation 54
CD58: cluster of differentiation 58
CIS: clinically isolated syndrome
CNS: central nervous system
HLA DRB1*15: HLA class II histocompatibility antigen, DRB1 β chain *15
HWE: Hardy–Weinberg equilibrium
ICAM1: intercellular adhesion molecule 1
IL7R: interleukin 7 receptor
IL2RA: interleukin 2 receptor subunit α
MHC: major histocompatibility complex
MI: myocardial infarction
OR: odds ratio
PPMS: primary progressive MS
RA: rheumatoid arthritis
RRMS: elapsing-remitting MS
SPMS: secondary progressive MS
WOS: Web of Science
