# Supplementary material for: The genetic effect of the ICAM1 (intercellular adhesion molecule 1) rs5498 polymorphism on the susceptibility towards multiple sclerosis
Source: Biosci Rep. 2018 Dec 11;38(6):BSR20181642. doi: 10.1042/BSR20181642 (PMC6294617; doi:10.1042/BSR20181642)
Supplement: Supplementary file 1 [file bsr20181642_Supp1.pdf]

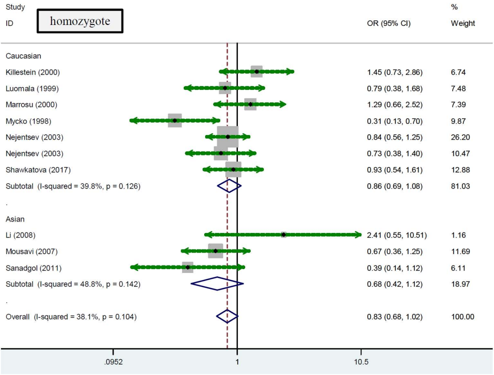
Study
ID
homozygote
OR (95% CI)
%
Weight
Caucasian
Killestein (2000) 1.45 (0.73, 2.86) 6.74
Luomala (1999) 0.79 (0.38, 1.68) 7.48
Marrosu (2000) 1.29 (0.66, 2.52) 7.39
Mycko (1998) 0.31 (0.13, 0.70) 9.87
Nejentsev (2003) 0.84 (0.56, 1.25) 26.20
Nejentsev (2003) 0.73 (0.38, 1.40) 10.47
Shawkatova (2017) 0.93 (0.54, 1.61) 12.88
Subtotal (I-squared = 39.8%, p = 0.126) 0.86 (0.69, 1.08) 81.03
Asian
Li (2008) 2.41 (0.55, 10.51) 1.16
Mousavi (2007) 0.67 (0.36, 1.25) 11.69
Sanadgol (2011) 0.39 (0.14, 1.12) 6.11
Subtotal (I-squared = 48.8%, p = 0.142) 0.68 (0.42, 1.12) 18.97
Overall (I-squared = 38.1%, p = 0.104) 0.83 (0.68, 1.02) 100.00
.0952
1
10.5

Figure S1

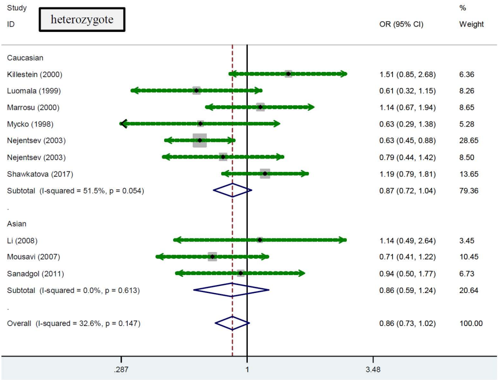

Study
ID
heterozygote
OR (95% CI)
%
Weight
Caucasian
Killestein (2000)
1.51 (0.85, 2.68)
6.36
Luomala (1999)
0.61 (0.32, 1.15)
8.26
Marrosu (2000)
1.14 (0.67, 1.94)
8.65
Mycko (1998)
0.63 (0.29, 1.38)
5.28
Nejentsev (2003)
0.63 (0.45, 0.88)
28.65
Nejentsev (2003)
0.79 (0.44, 1.42)
8.50
Shawkatova (2017)
1.19 (0.79, 1.81)
13.65
Subtotal (I-squared = 51.5%, p = 0.054)
0.87 (0.72, 1.04)
79.36
Asian
Li (2008)
1.14 (0.49, 2.64)
3.45
Mousavi (2007)
0.71 (0.41, 1.22)
10.45
Sanadgol (2011)
0.94 (0.50, 1.77)
6.73
Subtotal (I-squared = 0.0%, p = 0.613)
0.86 (0.59, 1.24)
20.64
Overall (I-squared = 32.6%, p = 0.147)
0.86 (0.73, 1.02)
100.00
.287
1
3.48

Figure S2

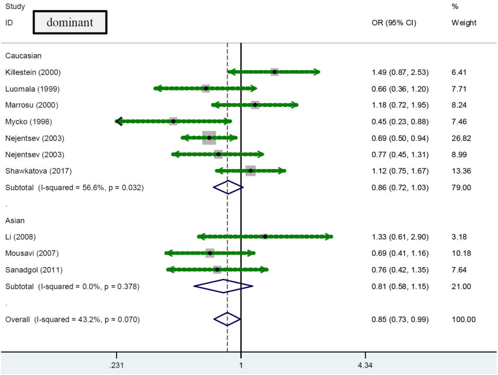
Study
ID
dominant
OR (95% CI)
%
Weight
Caucasian
Killestein (2000) 1.49 (0.87, 2.53) 6.41
Luomala (1999) 0.66 (0.36, 1.20) 7.71
Marrosu (2000) 1.18 (0.72, 1.95) 8.24
Mycko (1998) 0.45 (0.23, 0.88) 7.46
Nejentsev (2003) 0.69 (0.50, 0.94) 26.82
Nejentsev (2003) 0.77 (0.45, 1.31) 8.99
Shawkatova (2017) 1.12 (0.75, 1.67) 13.36
Subtotal (I-squared = 56.6%, p = 0.032) 0.86 (0.72, 1.03) 79.00
.
Asian
Li (2008) 1.33 (0.61, 2.90) 3.18
Mousavi (2007) 0.69 (0.41, 1.16) 10.18
Sanadgol (2011) 0.76 (0.42, 1.35) 7.64
Subtotal (I-squared = 0.0%, p = 0.378) 0.81 (0.58, 1.15) 21.00
.
Overall (I-squared = 43.2%, p = 0.070) 0.85 (0.73, 0.99) 100.00
.231
1
4.34

Figure S3

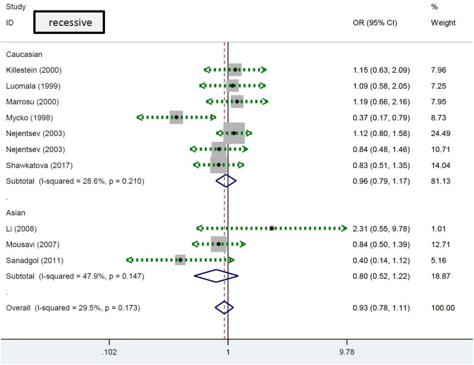

Study
ID
recessive
OR (95% CI)
%
Weight
Caucasian
Killestein (2000)
1.15 (0.63, 2.09)
7.96
Luomala (1999)
1.09 (0.58, 2.05)
7.25
Marrosu (2000)
1.19 (0.66, 2.16)
7.95
Mycko (1998)
0.37 (0.17, 0.79)
8.73
Nejentsev (2003)
1.12 (0.80, 1.58)
24.49
Nejentsev (2003)
0.84 (0.48, 1.46)
10.71
Shawkatova (2017)
0.83 (0.51, 1.35)
14.04
Subtotal (I-squared = 28.6%, p = 0.210)
0.96 (0.79, 1.17)
81.13
.
Asian
Li (2008)
2.31 (0.55, 9.78)
1.01
Mousavi (2007)
0.84 (0.50, 1.39)
12.71
Sanadgol (2011)
0.40 (0.14, 1.12)
5.16
Subtotal (I-squared = 47.9%, p = 0.147)
0.80 (0.52, 1.22)
18.87
.
Overall (I-squared = 29.5%, p = 0.173)
0.93 (0.78, 1.11)
100.00
.102
1
9.78

Figure S4

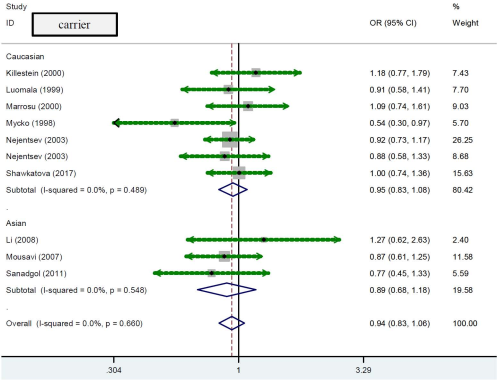
Study
ID
carrier
OR (95% CI)
%
Weight
Caucasian
Killestein (2000) 1.18 (0.77, 1.79) 7.43
Luomala (1999) 0.91 (0.58, 1.41) 7.70
Marrosu (2000) 1.09 (0.74, 1.61) 9.03
Mycko (1998) 0.54 (0.30, 0.97) 5.70
Nejentsev (2003) 0.92 (0.73, 1.17) 26.25
Nejentsev (2003) 0.88 (0.58, 1.33) 8.68
Shawkatova (2017) 1.00 (0.74, 1.36) 15.63
Subtotal (I-squared = 0.0%, p = 0.489) 0.95 (0.83, 1.08) 80.42
.
Asian
Li (2008) 1.27 (0.62, 2.63) 2.40
Mousavi (2007) 0.87 (0.61, 1.25) 11.58
Sanadgol (2011) 0.77 (0.45, 1.33) 5.59
Subtotal (I-squared = 0.0%, p = 0.548) 0.89 (0.68, 1.18) 19.58
.
Overall (I-squared = 0.0%, p = 0.660) 0.94 (0.83, 1.06) 100.00
.304
1
3.29

Figure S5

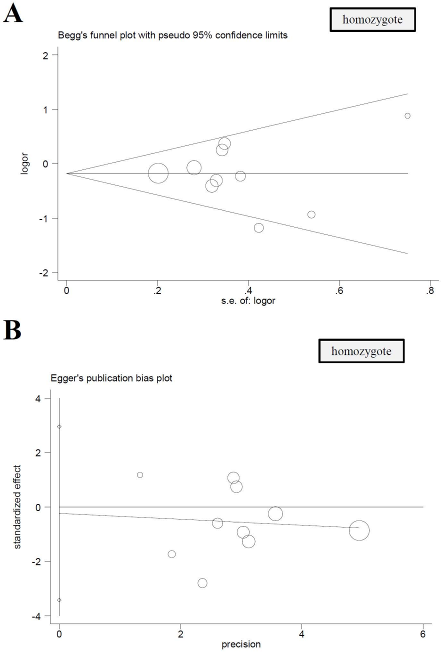
A
homozygote
Begg's funnel plot with pseudo 95% confidence limits
logor
2
1
0
-1
-2
0
.2
.4
.6
.8
s.e. of: logor
B
homozygote
Egger's publication bias plot
standardized effect
4
2
0
-2
-4
0
2
4
6
precision

Figure S6

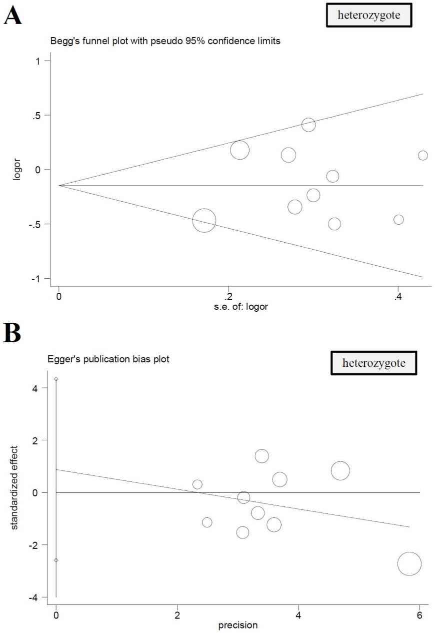

A
heterozygote
Begg's funnel plot with pseudo 95% confidence limits
1
.5
0
-.5
-1
logor
0
.2
.4
s.e. of: logor
B
Egger's publication bias plot
heterozygote
4
2
0
-2
-4
standardized effect
0
2
4
6
precision

Figure S7

A

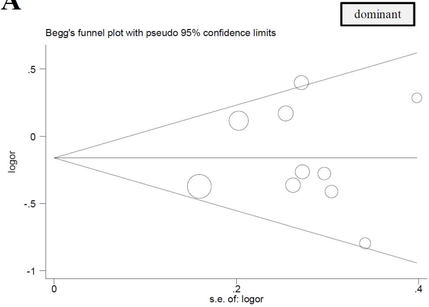
dominant
Begg's funnel plot with pseudo 95% confidence limits
.5
0
-.5
-1
logor
0
.2
.4
s.e. of: logor

B

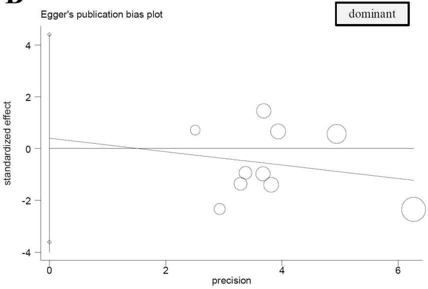
Egger's publication bias plot
dominant
4
2
0
-2
-4
standardized effect
0
2
4
6
precision

Figure S8

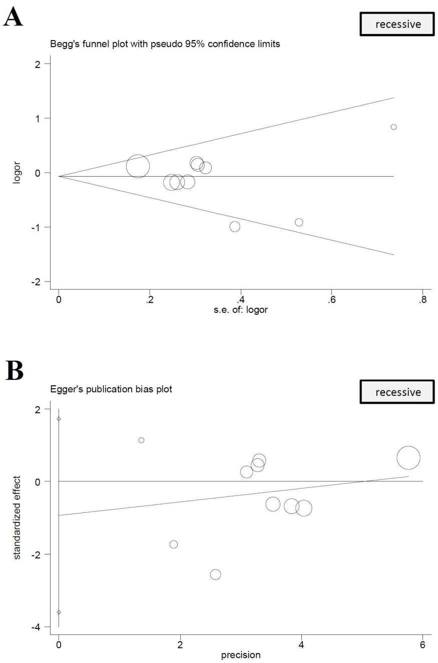
A
recessive
Begg's funnel plot with pseudo 95% confidence limits
logor
2
1
0
-1
-2
0
.2
.4
.6
.8
s.e. of: logor
B
Egger's publication bias plot
recessive
standardized effect
2
0
-2
-4
0
2
4
6
precision

Figure S9

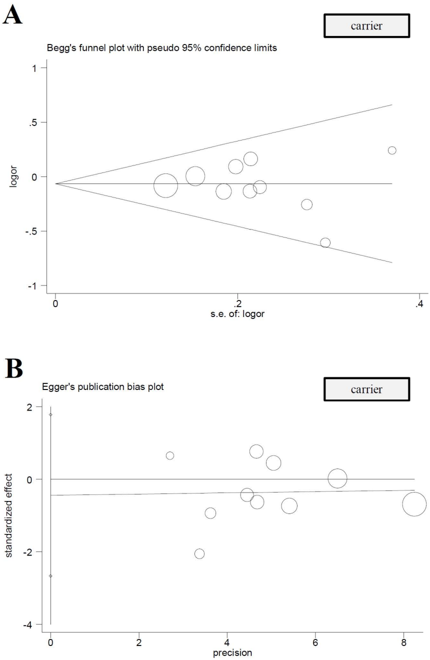
A
carrier
Begg's funnel plot with pseudo 95% confidence limits
logor
s.e. of: logor
1
.5
0
-.5
-1
0
.2
.4
B
carrier
Egger's publication bias plot
standardized effect
precision
2
0
-2
-4
0
2
4
6
8

Figure S10
